# Minute‐Scale Repeated Metabolic Probing of Living Cells Enabled by Rapid Parahydrogen‐Based Hyperpolarization

**DOI:** 10.1002/anie.4855536

**Published:** 2026-07-10

**Authors:** Philipp R. Groß, Stefan Petersen, Zirun Wang, Behnam Shamshiri, Henri de Maissin, Sebastian Lucas, Oliver Gorka, Lisa Heß, Irene Marco‐Rius, Lluís Mangas‐Florencio, André F. Martins, Philipp Boehm‐Sturm, Maxim Zaitsev, Robert Zeiser, Jan‐Bernd Hövener, Thomas Reinheckel, Olaf Groß, Andreas B. Schmidt

**Affiliations:** ^1^ Division of Medical Physics Department of Radiology Medical Center and Faculty of Medicine Freiburg Germany; ^2^ German Cancer Consortium (DKTK), Partner Site Freiburg a Partnership Between DKFZ and University Medical Center Freiburg Heidelberg Germany; ^3^ Albert Ludwigs University Freiburg Faculty of Biology Freiburg Germany; ^4^ NVision Imaging Technologies GmbH Ulm Germany; ^5^ Faculty of Medicine Medical Center Institute of Neuropathology University of Freiburg Freiburg Germany; ^6^ Institute of Molecular Medicine and Cell Research Faculty of Medicine University of Freiburg Freiburg Germany; ^7^ Institute For Bioengineering of Catalonia (IBEC) Barcelona Institute of Science and Technology (BIST) Barcelona Spain; ^8^ University of Barcelona Barcelona Spain; ^9^ Department of Preclinical Imaging and Radiopharmacy Werner Siemens Imaging Center Eberhard Karls University Tübingen Tübingen Germany; ^10^ Cluster of Escellence iFIT (EXC 2180) “Image‐Guided and Functionally Instructed Tumor Therapies” University of Tübingen Tübingen Germany; ^11^ Charité 3R – Replace, Reduce, Refine Charité – Universitätsmedizin Berlin, Corporate Member of Freie Universität Berlin and Humboldt‐Universität zu Berlin Berlin Germany; ^12^ Department of Experimental Neurology and Center for Stroke Research Berlin Charité–Universitätsmedizin Berlin Berlin Germany; ^13^ Charité Core Facility Experimental MRIs (RRID SCR_017810) Charité – Universitätsmedizin Berlin Berlin Germany; ^14^ German Cancer Consortium (DKTK) Partner Site Berlin, a Partnership Between DKFZ and Charité – Universitätsmedizin Berlin Berlin Germany; ^15^ Faculty of Medicine Hematology and Oncology Department of Medicine I Medical Center University of Freiburg Freiburg Germany; ^16^ Center For Integrative Biological Signalling Studies (CIBSS) University of Freiburg Freiburg Germany; ^17^ Sektion Biomedizinsche Bildgebung Molecular Imaging North Competence Center MOINCC Klinik Für Radiologie und Neuroradiologie University Hospital Schleswig‐Holstein Kiel University Kiel Germany; ^18^ Centre for Biological Signalling Studies (BIOSS) University of Freiburg Freiburg Germany

**Keywords:** bioreactor, carbon‐13, hyperpolarization, metabolism, parahydrogen, pyruvate, SABRE

## Abstract

Hyperpolarized (HP) ^13^C nuclear magnetic resonance (NMR) spectroscopy enables real‐time observation of metabolic fluxes but is typically limited to single‐shot measurements due to complex preparation procedures and low experimental throughput. Here, we established a rapid and experimentally accessible workflow for temporally controlled HP measurements in living cells. Using SABRE‐SHEATH at 0.4 µT, we achieved 7.7% ± 0.2% ^13^C polarization of 50 mM [1‐^13^C]pyruvate within 60 s. Simple 1:50 dilution with phosphate‐buffered D_2_O yielded cell‐compatible solutions that retained 5.3% ± 0.4% polarization of 1.3 mM pyruvate without multi‐step purification. Combined with a simplified agarose bead immobilization approach, this enabled four injections of HP pyruvate into the same HeLa cell population within 7 min. Under rapid (≈2 min) reinjection intervals, the pyruvate‐to‐lactate conversion progressively declined, whereas stable metabolic conversion was maintained at 20 min intervals with intermittent cell medium perfusion. These findings demonstrate that rapid repeated substrate delivery can transiently exhaust cellular metabolic conversion capacity. This experimentally accessible operating mode enables the study of short‐term metabolic dynamics that remain obscured in conventional single‐shot HP‐NMR or long timescale thermally polarized NMR measurements.

## Graphical abstract

Rapid parahydrogen hyperpolarization combined with simple dilution enables biocompatible delivery of hyperpolarized pyruvate to immobilized living cells within ≈1 min. Repeated substrate injections reveal frequency‐dependent metabolic dynamics, introducing a new temporal dimension for hyperpolarized NMR studies of cellular metabolism and enabling rapid probing of short‐term metabolic responses.

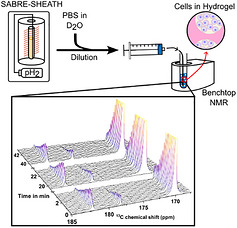

## 1 Introduction

Metabolic reprogramming is a hallmark of cancer and provides invaluable insight into tumor biology, therapeutic vulnerabilities, and treatment response [1, 2]. Understanding how metabolic fluxes dynamically respond to acute perturbations is central for dissecting causal mechanisms and guiding therapeutic strategies in living systems. While several analytical and imaging approaches can track metabolic responses indirectly, direct, non‐invasive quantification of pathway‐specific fluxes under controlled and repeated substrate stimulation in the same living cell population remains largely inaccessible [3, 4].

Nuclear magnetic resonance spectroscopy (NMR) or magnetic resonance imaging (MRI) of hyperpolarized (HP) metabolites, however, hold the potential to address this matter. Hyperpolarization enhances NMR spectroscopy and MRI sensitivity by more than four orders of magnitude, by increasing nuclear spin polarization [5]. This dramatic signal enhancement enables the detection of low‐abundance ^13^C‐labeled metabolites and their enzymatic conversion in real time. HP magnetic resonance (MR) uniquely allows direct and non‐invasive observation of metabolic fluxes in vitro and in vivo without ionizing radiation [6, 7, 8].

A key limitation of all hyperpolarization approaches, however, is the short lifetime of the polarization (typically ≈1 min), which severely restricts the accessible observation window. Despite promising approaches to prolong polarization lifetimes [9], this remains the most significant hurdle for HP MR. As a result, monitoring metabolic conversions and reactions to stimuli exceeding this period has not been possible so far.

Existing hyperpolarization strategies have either lacked cell compatibility or the ability to efficiently hyperpolarize metabolites of interest [10, 11], or have required preparation times incompatible with rapid repeated perturbation experiments [12, 13, 14, 15]. Approaches to overcome this limitation have included parallelized hyperpolarization (e.g., multi‐sample dissolution Dynamic Nuclear Polarization (dDNP)) [16, 17], ^1^H‐^13^C cross‐polarization dDNP [18], or fast parahydrogen (pH_2_)‐based hyperpolarization [11, 19, 20], which can enable quasi‐continuous production of highly HP ^13^C molecules [10]. pH_2_‐based hyperpolarization methods appear particularly interesting, as they are orders of magnitude faster than other techniques.

In the Signal Amplification by Reversible Exchange (SABRE) method, pH_2_ and a substrate reversibly bind to an iridium N‐heterocyclic carbene complex, enabling coherent transfer of pH_2_ spin order to heteronuclei of the substrate without chemical modification [21]. Since the development of SABRE‐SHEATH (SABRE in Shield Enables Alignment Transfer to Heteronuclei) [14], and its first successful demonstration for pyruvate hyperpolarization [12], substantial progress has been made in catalyst design [22], large‐scale catalyst production procedures [23, 24], spin‐order transfer schemes [25], and experimental optimization [26, 27, 28, 29, 30], yielding steadily increasing ^13^C polarization levels [26, 31]. Notably, recent pioneering studies have demonstrated the application of SABRE‐hyperpolarized [1‐^13^C]pyruvate and [1‐^13^C]ketoleucine for metabolic investigations in living cells using compact benchtop NMR systems, including measurements in suspension cultures of yeast cells, highlighting the potential of SABRE for rapid and accessible cellular metabolic studies [32, 33]. Importantly, SABRE enables polarization within seconds to minutes.

Despite these advances, rapid and repeated polarization of metabolically relevant substrates such as pyruvate has remained challenging within parahydrogen‐based approaches. More broadly, translating any hyperpolarization strategy into a practical high‐throughput workflow enabling minute‐scale repeated metabolic probing in living cells has not yet been achieved.

A severe challenge toward this goal is the generation of biocompatible solutions, which typically requires multi‐step purification procedures, including catalyst scavenging, phase separation, or solvent evaporation, each necessitating substrate‐specific optimization [34, 35]. While these methods enabled the first SABRE‐based in vivo ^13^C MRI studies, they introduce preparation time and procedural complexity that prevent rapid, iterative experimentation [34, 36, 37, 38, 39]. Consequently, the intrinsic speed advantage of pH_2_ hyperpolarization has not yet been fully leveraged for high‐frequency perturbation studies in cellular systems.

Here, we present methods for producing HP pyruvate for cellular applications repeatedly every 2 min. We introduce a simple dilution‐based strategy for generating biocompatible HP solutions for in cellulo experiments directly from pH_2_‐HP substrates, demonstrated here using SABRE‐SHEATH and [1‐^13^C]pyruvate. This becomes feasible because hyperpolarization is performed at substrate concentrations far above those needed for cellular studies, allowing simple dilution to biocompatible conditions without multi‐step purification.

For testing this novel procedure in living cells, we were faced with a second problem: sedimentation and spatial inhomogeneity of suspended cells can complicate quantitative interpretation, as the effective number of cells within the sensitive detection volume may vary over time. In parallel, in oncological research, increasing attention has been devoted to immobilized as well as two‐ and three‐dimensional culture systems, which offer improved spatial stability and, in many cases, more physiologically relevant microenvironments [40, 41, 42, 43, 44]. Building on these developments, experimentally straightforward formats that facilitate rapid and repeated HP substrate delivery under defined temporal conditions represent a particularly attractive next step [45, 46, 47, 48, 49, 50, 51].

To address this, we developed a straightforward agarose bead immobilization method that ensures stable positioning of living cells within the NMR detection volume and allows for continuous medium delivery without loss of cells from the sample.

Together, the rapid production of HP pyruvate and innovative NMR cell culture enabled, for the first time, reproducible repeated delivery of HP metabolites at minute‐scale intervals to the same cellular population. This operational mode introduces a fundamentally new experimental timescale for HP NMR, enabling the investigation of short‐term metabolic responses that remain inaccessible in conventional single‐injection measurements.

## 2 Results and Discussion

### 2.1 Rapid Generation of Biocompatible Hyperpolarized Pyruvate by Dilution

**FIGURE 1:**
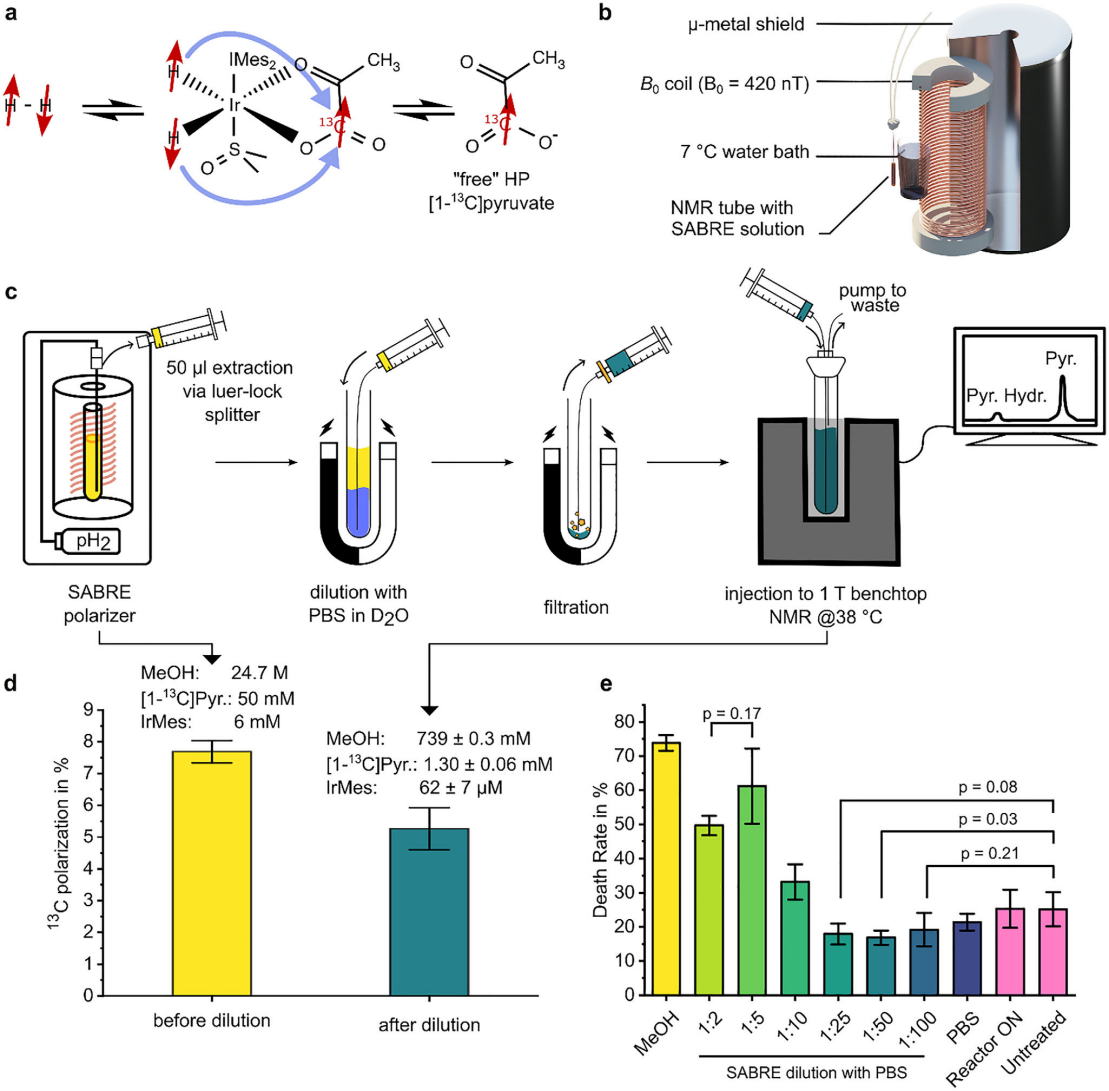
(a) Schematic representation of the reversible exchange of parahydrogen and [1‐^13^C]pyruvate on the Ir‐catalyst exploited to transfer spin order from the hydrogen atoms to the 1‐^13^C label in the pyruvate. (b) Schematic SABRE setup consisting of a B_0_ coil and sample held in a 5 mm NMR tube placed in a temperature‐controlled water bath inside the magnetic shield. (c) Schematic workflow of the experimental procedure consisting of extraction of 50 µL SABRE solution from the polarizer after 60 s of polarization, 1:50 dilution with PBS in D_2_O in an elevated magnetic field followed by filtration and subsequent injection to the NMR tube, held in a 1 T benchtop NMR spectrometer set to 38°C. (d) ^13^C polarization levels achieved through SABRE‐SHEATH polarization and after subsequent dilution and filtration. Error bars indicate standard errors (SE). (e) Death rate of agarose‐embedded HeLa cells after treatment with the specified conditions for 3 × 3 min with 5 min intermediate culture‐medium exposition (simulating 3 administrations of MeOH, PBS, or mixtures) and after 18 h in the bioreactor at 37°C; or of an untreated control (Figure S3). The death rate was evaluated using PI and Hoechst33342 staining followed by confocal microscopy and image analysis in ImageJ. Error bars indicate SE for *N* = 5 (1:2 and 1:5 dilutions) or *N* = 13 independent experiments. For clarity, two‐sample *t*‐tests were performed (OriginPro 2024) for selected conditions to assess apparent variability. A small but statistically significant difference was observed between the 1:50 condition and the untreated control (*p* = 0.03), although this effect is not part of a consistent trend, as no significant differences were observed for other dilution levels (e.g., 1:25 and 1:100 vs. untreated).

Hyperpolarization of [1‐^13^C]pyruvate was performed using SABRE‐SHEATH at 0.4 µT (Figure 1a). The experimental setup required only a three‐layer mu‐metal shielding, a field‐generating coil, a temperature‐controlled water bath, and an NMR tube containing 6 mM IMes catalyst, produced according to Savka et al. [52] (see Supporting Information), 50 mM pyruvate, 30 mM DMSOd_6_ and 0.65 mM EDTA in 600 µL methanol, through which pH_2_ (≈90% enrichment) was guided during SABRE (Figure 1b). Under optimized conditions, ^13^C polarization levels of 7.7% ± 0.2% (*N* = 4; see Table S1) were achieved for 50 mM pyruvate in methanol within 60 s (Figure 1d).

For cellular studies, substantially lower pyruvate concentrations (< 5 mM) are generally considered optimal to avoid excessive substrate loading and potential saturation of transmembrane transporters or lactate dehydrogenase (LDH) [53, 54]. Exploiting the fact that SABRE efficiently polarizes pyruvate at much higher concentrations, we chose a simple dilution step to produce an HP solution with the desired pyruvate concentration. For this, 50 µL of the HP SABRE solution was directly diluted 1:50 with phosphate‐buffered saline (PBS) in D_2_O. As partial catalyst precipitation occurred during dilution, the solution was passed through a 1.2 µm filter during syringe loading to eliminate any solids and further reduce the catalyst content. Throughout the procedure, the sample was maintained at 120 mT to prolong the hyperpolarization lifetime (Figure 1c, Figure S1), which relaxed with the longitudinal relaxation time *T*_1_. Analysis of the resulting 2.5 mL solution revealed that the final 1.3 ± 0.06 mM pyruvate retained a polarization of 5.3% ± 0.4% (*N* = 4; Figure 1d), sufficient for detection in subsequent cellular experiments.

Dilution and filtration also reduced methanol and catalyst concentrations to approximately 739 ± 30 mM and 62 ± 7 µM, respectively (*N* = 6; Table S1 and Figure S2). Notably, in our previous study, comparable methanol and catalyst concentrations did not significantly affect cultured cell viability even after 48 h exposure [34]. To assess our assumption that our produced solution was biocompatible, we additionally evaluated the viability of our embedded cells across a range of dilution factors using propidium iodide (PI) and Hoechst 33342 staining, confocal microscopy, and image analysis with ImageJ (Figure 1e, Figure S3). At high dilution ratios, a small but statistically significant difference was observed between the 1:50 condition and the untreated control (*p* = 0.03), although this effect is not part of a consistent trend, as no significant differences were observed for other dilution levels (e.g., 1:25 and 1:100 vs. untreated). This isolated observation is therefore not further interpreted and is likely attributable to statistical variation. Considering that, in our HP‐NMR experiments, cells are exposed only briefly to the injected HP substrate bolus (≈2–7 min), followed by immediate washing and replenishment with fresh cell medium, we concluded that direct dilution provides a practical route to biocompatible solutions for first in cellulo applications. Together, these results demonstrate that high‐concentration SABRE polarization combined with direct dilution enables rapid generation of cell‐compatible HP pyruvate without multi‐step purification. This simplified preparation removes a major procedural bottleneck and enables rapid iteration of pH_2_‐based hyperpolarization workflows.

### 2.2 Immobilization of Cells in Agarose

Repeated perturbation of the same cellular population requires spatial stability within the sensitive detection volume of the NMR probe. Cell suspensions are prone to sedimentation and positional variability, which can lead to fluctuations in the effective cell number within the detection region and, consequently, to signal instability and reduced reproducibility. Moreover, repeated measurements in suspension often require additional handling steps, such as removal and reconstitution of the cell medium [55], which complicate short repetition intervals and can compromise experimental consistency and cell viability.

**FIGURE 2:**
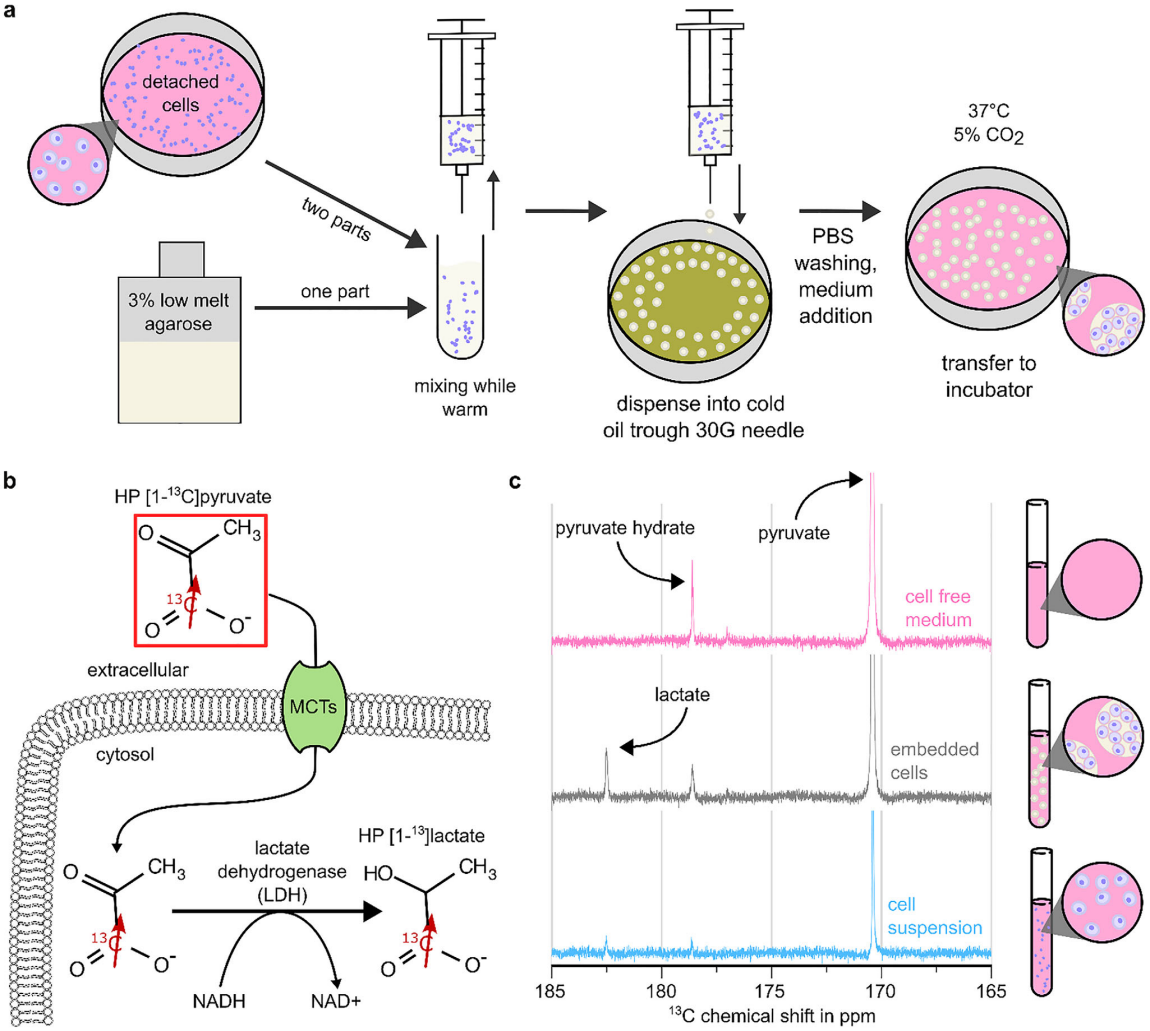
(a) Schematic workflow of cell embedding in low‐melt agarose: detached cells are mixed with molten agarose, dispensed through a 30G needle into cold sunflower oil to form beads, followed by PBS washing and transfer to culture medium. (b) Schematic of HP [1‐^13^C]pyruvate transport into the cell via monocarboxylate transporters (MCT) and its enzymatic conversion to [1‐^13^C]lactate by lactate dehydrogenase, accompanied by oxidation of NADH to NAD+. (c) ^13^C NMR spectra acquired using a 90° pulse 20 s after identically prepared HP pyruvate injections (composed as in Figure 1d) into cell‐free medium, agarose‐embedded cells, and cell suspensions (7.5 × 10^6^ cells each).

To overcome this limitation, we developed a simple cell immobilization and bioreactor strategy (Figure 2a; Figures S5, S6), inspired by previously published work [45, 46, 50, 51, 56, 57]. Detached cells were mixed with low‐melt agarose and dispensed into cold oil through a 30 G needle, forming uniform agarose beads with an average diameter of ≈1.93 mm (*N* = 23) and an average cell count of ≈2.5·× 10^5^ cells per bead. After PBS washing and medium exchange, the beads were kept in standard culture conditions prior to HP experiments.

### 2.3 HP Experiments With Agarose Embedded HeLa

After confirming polarization retention throughout dilution and validating the biological compatibility of our approach, we proceeded to inject our HP [1‐^13^C]pyruvate solution into 7.5 × 10^6^ HeLa cells (either suspended or hydrogel‐embedded), as well as into plain cell culture medium, inside the NMR bore set to a temperature of 38°C, to compare the occurrence of downstream metabolic products in each sample. HP ^13^C NMR spectra acquired 20 s after injection demonstrated high‐SNR [1‐^13^C]pyruvate and [1‐^13^C]lactate signals in both embedded cells and cells in suspension; as expected, no lactate production was detected in the cell‐free medium (Figure 2c). Notably, while lactate production was observed in both cell‐containing samples, the lactate/pyruvate ratio was higher for embedded cells (0.092) than for suspended cells (0.062).

We attribute this difference to several factors. As HeLa cells are inherently adherent, a lack of extracellular matrix (ECM) and adhesion cues may cause metabolic alterations compared to native status in both experiment configurations. Additionally, substrate diffusion, local cell density, and substrate availability can differ between the experiment settings. Furthermore, agarose can limit nutrient diffusion [58]. Additionally, upon injection of the diluted SABRE solution into the NMR tube containing suspended cells, the total volume may exceed the sensitive region of the RF coil (Figure S8), such that only a fraction of the metabolically active cells contributes effectively to the detected lactate signal. In addition, in this configuration the injected SABRE solution is diluted approximately 2‐fold in the existing cell suspension, reducing the effective pyruvate concentration available for cellular uptake and conversion. In contrast to cell suspensions, the agarose‐embedded cells maintain a defined spatial distribution within the active NMR volume. Upon injection, the surrounding medium is transiently replaced by the HP solution, while the immobilized cells remain confined to the detection region. This configuration yields a more static and reproducible metabolic environment without requiring mechanical mixing, which would otherwise interfere with kinetic ^13^C NMR signal acquisition. These features are essential for enabling repeated injections at controlled temporal intervals and meaningful results. However, the use of agarose or other matrices introduces potential limitations related to substrate transport by diffusion [58, 59]. Delivery to cells in the interior of the construct occurs on longer timescales and may result in transient concentration gradients. In combination with cellular consumption at the periphery, this can lead to spatially heterogeneous substrate availability and complicate data interpretation and modeling. While the robust lactate signals observed in our experiments, particularly compared to suspension‐based measurements, indicate that a substantial fraction of cells is reached by the injected substrate and actively metabolizes it, complete and homogeneous substrate distribution throughout the bead cannot be assumed. This is especially relevant given the relatively large bead diameter used here (≈1.9 mm), for which full equilibration on the timescale of the experiment is unlikely. A potential strategy to mitigate these effects is the use of smaller bead diameters [51], which could be achieved with our method using finer syringe cannulas and will be explored in future work.

To assess the compatibility of the agarose bead setting for extended experimental conditions, the viability of embedded cells maintained in the bioreactor was evaluated. Cells remained viable after overnight incubation (18 h) at 37°C under continuous medium circulation (≈55 µL·min^−1^) as well as after treatment with ≤ 1:25‐diluted SABRE solution for 3 × 3 min with intermediate culture medium exposure (5 min), confirming that the immobilization and perfusion conditions do not compromise cellular viability (Figure 1e).

Overall, agarose bead immobilization establishes stable, defined experimental conditions for controlled metabolic measurements in living cells.

### 2.4 Frequency‐Dependent Metabolic Response Under Repeated Hyperpolarized Bolus Administration

**FIGURE 3:**
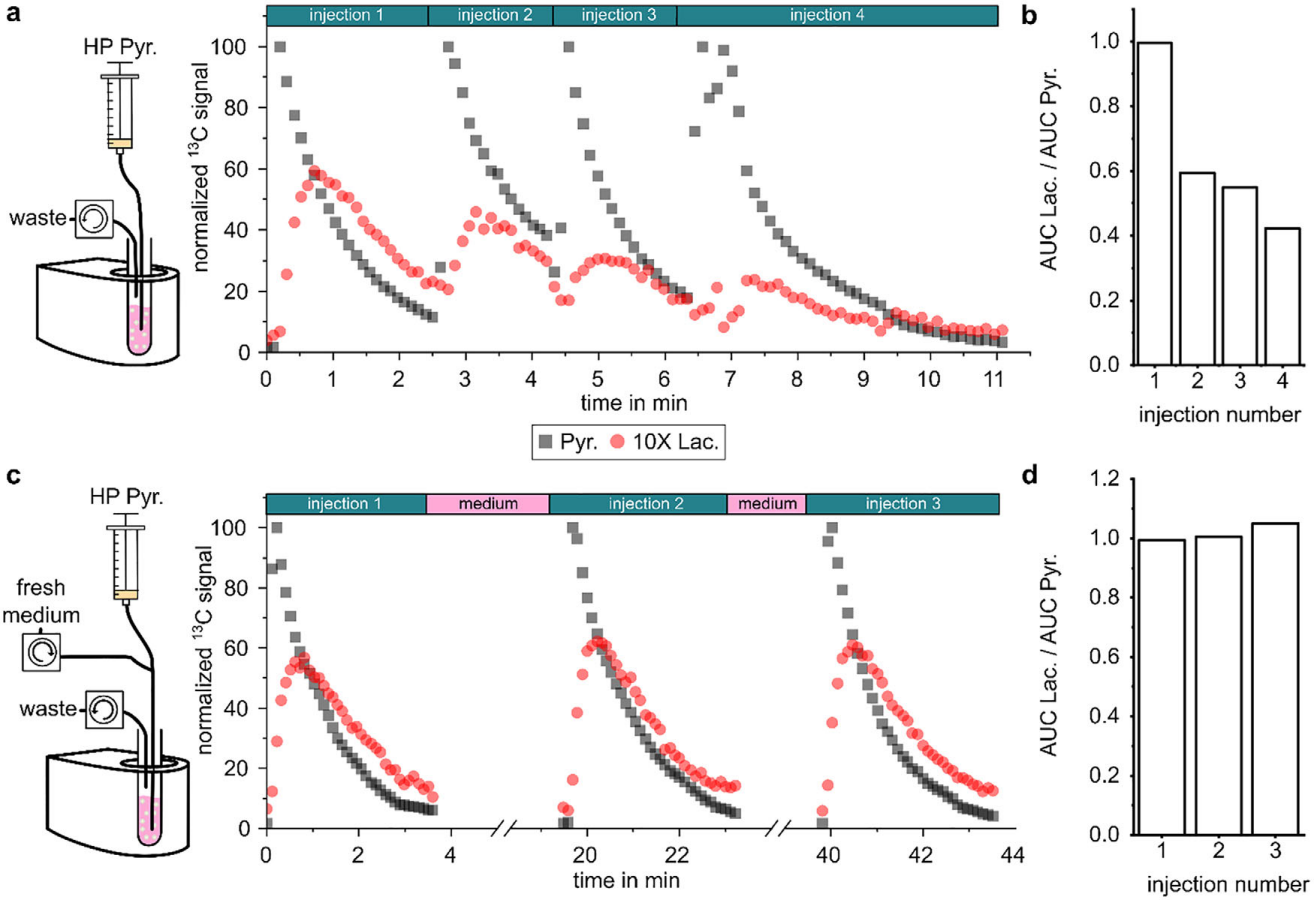
Integrated ^13^C NMR signals of pyruvate (Pyr.) and lactate (Lac.) dynamically recorded following repeated injection of HP [1‐^13^C]pyruvate into hydrogel‐embedded HeLa cells (7.5 × 10^6^ cells) with repetition times of ≈2 min (a) and ≈20 min (c). Between the injections in (c), the embedded cells were perfused with fresh cell medium. The data for each injection was normalized to the respective maximum pyruvate signal to support comparing the lactate dynamics. The recorded ^13^C NMR spectra are shown in Figure S7. Panels (b) and (d) display the lactate‐to‐pyruvate AUC ratios derived from the dynamic data shown in panels a and c, respectively.

We next combined rapid SABRE polarization with repeated injections into the same immobilized HeLa cell populations. Two injection regimes were investigated: rapid repetition at 2 min intervals (Figure 3a,b) and delayed repetition at 20 min intervals (Figure 3c,d). For the latter condition, cells were perfused with fresh medium for about 15 min between successive measurements.

Under rapid repetition (Δ*t* ≈ 2 min), lactate production progressively declined across successive pyruvate injections as reflected by decreasing lactate‐to‐pyruvate ratios of the time‐integrated signals (area under the curve, AUC; Figure 3b). In contrast, under delayed injection conditions (Δ*t* ≈ 20 min), lactate production remained stable across the repetitions. The lactate‐to‐pyruvate AUC ratios were consistent, indicating preservation of metabolic conversion capacity (Figure 3d).

These findings demonstrate that repeated delivery of HP substrates can induce an injection‐interval‐dependent metabolic response. The observed decline in lactate production under rapid perturbation is consistent with a transient limitation in metabolic capacity, potentially reflecting depletion of reducing equivalents such as NADH, transporter saturation, or enzymatic throughput constraints (Figure 2b). We further support this hypothesis over a persistent or dominant impurity‐related effect (MeOH or iridum) on metabolic conversion, as lactate production recovers to baseline levels after 15 min of medium perfusion following repeated HP pyruvate injections (Figure S4). This is further supported by the observation that under longer waiting intervals, the AUC ratios remain stable without detectable drift (Figure 3c,d). In particular, under rapid repeated injection conditions, a temporary imbalance in the cellular NADH/NAD^+^ redox state may contribute to the reduced conversion efficiency, consistent with previous reports on redox‐dependent modulation of lactate production [60], although this requires further investigation

The recovery observed at longer intervals, and the absence of declining lactate production when injections were separated by a 15 min medium perfusion phase, suggest that metabolic homeostasis is restored over minutes and support the interpretation that the reduced conversion under rapid repetition reflects a reversible metabolic constraint rather than an experimental artifact.

Importantly, sample preparation and injection protocols were identical across both regimens, isolating temporal frequency as the determining variable. These results demonstrate that rapid, repeated HP substrate delivery enables experimental access to short‐timescale metabolic capacity limits and recovery processes that are difficult to probe with conventional single‐shot hyperpolarization approaches.

HP ^13^C NMR experiments have traditionally been conceptualized as sensitivity‐enhanced snapshots of metabolic conversion. The present work introduces a complementary operational mode: rapid, repeated delivery of HP metabolites that allows controlled variation in injection intervals within the same cellular population. By combining fast parahydrogen‐based polarization, direct dilution to biocompatible conditions, and spatially stable cell immobilization, we enable minute‐scale reinjection under well‐defined experimental conditions.

In contrast to prior approaches based on suspended cells or post‐injection drying procedures and building on earlier immobilization concepts reported for HP NMR studies [45, 46, 47], our agarose bead configuration maintains spatial stability and viability while permitting repeated substrate exposures. This stability is essential to distinguish genuine metabolic adaptation from signal variability caused by cell redistribution or changes in the effective cell number within the detection volume.

The frequency‐dependent attenuation of lactate production observed under rapid reinjection conditions reveals an additional dimension of HP‐NMR measurements: the emergence of metabolic capacity limits under repeated substrate exposure. While a detailed mechanistic dissection lies beyond the scope of this study, the data appear consistent with transient constraints in redox availability or transport processes. The ability to probe such limitations in real time extends the conceptual scope of HP NMR beyond single‐time‐point observation to the investigation of dynamic metabolic responses under temporally controlled experimental conditions.

We demonstrated the dilution‐based workflow using SABRE‐HP pyruvate prepared in methanol, which we refer to as “SABRE‐dilute.” However, this approach is not limited to this substrate, medium, or polarization method. Parahydrogen‐based induced polarization (PHIP) methods, including hydrogenative PHIP (“PHIP‐dilute”), are inherently compatible with rapid polarization cycles and high substrate concentrations [10, 61, 62, 63, 64, 65, 66, 67]. The presented approach may enable entirely new applications and may facilitate accelerated biological evaluation of newly polarized metabolites. Many parahydrogen‐HP compounds have remained confined to polarization feasibility studies due to purification constraints prior to cellular testing [68, 69, 70, 71]. The simplified workflow presented here lowers this barrier and can shorten the transition from polarization chemistry to first biological interrogations.

However, despite the short contact times and the absence of significant changes in viability in the present study, as well as in agreement with previous studies employing longer exposure times and metabolic MTT assays [34], an influence of residual methanol on cellular metabolism cannot be fully excluded. Importantly, even in the presence of a potential global metabolic effect, the presented approach remains sensitive to relative changes in metabolic conversion under controlled experimental conditions. Further reduction of residual solvent levels is nevertheless desirable and may be achieved by increasing substrate concentration during polarization. For example, sodium pyruvate exhibits higher solubility in methanol (≈80 mM) [26] than used in the present study (50 mM), which may potentially be further improved using co‐solvents [72]. In addition, PHIP‐SAH has demonstrated efficient preparation of highly concentrated agents (e.g., up to 1 M) [61, 73, 74]. Alternatively, solvent systems such as acetone–water mixtures (Ace‐SABRE) offer a promising route to further reduce residual solvent levels [39, 75].

Taken together, these developments establish rapid pH_2_ hyperpolarization as a practical approach for temporally resolved metabolic perturbation studies and enable iterative cellular experiments that are difficult to realize with slower polarization techniques. The method may be particularly valuable for preclinical metabolic screening, pharmacological pathway analysis, and the study of therapeutic interventions, as well as for applications in microfluidic or organ‐on‐chip systems [76, 77], where rapid and repeated metabolic interrogation is required.

## 3 Conclusion

In summary, this work establishes a rapid experimental operating mode for HP metabolic NMR by enabling the generation of cell‐compatible pH_2_‐polarized substrate boluses within approximately 1 min. This SABRE‐dilute strategy, combined with agarose bead immobilization, enables reproducible repeated delivery of HP metabolites to the same cellular population without complex purification procedures. This capability expands the conceptual scope of HP NMR beyond single‐time‐point metabolic snapshots toward probing dynamic metabolic regulation and response in living cellular systems. We anticipate that this experimentally accessible strategy will accelerate the adoption of pH_2_‐based hyperpolarization in preclinical metabolic research and enable new longitudinal applications, including the assessment of therapy response, pharmacological pathways, and studies in microfluidic and organ‐on‐chip platforms.

## Author Contributions

**Philipp R. Groß**: investigation, writing – original draft, writing – review and editing, methodology, visualization, validation, data curation, formal analysis, software. **Stefan Petersen**: investigation, writing – original draft, methodology, writing – review and editing, validation. **Zirun Wang**: methodology, writing – review and editing, data curation. **Behnam Shamshiri**: methodology. **Henri de Maissin**: validation, visualization, writing – review and editing, data curation. **Sebastian Lucas**: methodology, writing – review and editing, validation, investigation. **Oliver Gorka**: writing – review and editing, methodology. **Lisa Heß**: methodology, writing – review and editing. **Irene Marco‐Rius**: writing – review and editing. **Lluís Mangas‐Florencio**: writing – review and editing. **André F. Martins**: writing – review and editing. **Philipp Boehm‐Sturm**: writing review and editing. **Maxim Zaitsev**: writing – review and editing, resources. **Robert Zeiser**: writing – review and editing. **Jan‐Bernd Hövener**: writing – review and editing. **Thomas Reinheckel**: writing – review and editing, resources, supervision, writing – original draft. **Olaf Groß**: writing – review and editing, resources, supervision. **Andreas B. Schmidt**: conceptualization, funding acquisition, writing – original draft, writing – review and editing, validation, visualization, project administration, resources, supervision.

## Conflicts of Interest

Sebastian Lucas is an employee of NVision Imaging Technologies GmbH. A provisional patent application for the presented method was submitted. Other than that, the authors have no conflicts of interests to declare.

## Supporting information



Please find additional experimental details, sample characterization, and NMR data in the Supporting Information file. The authors have cited additional references within the Supporting Information [78, 79].
**Supporting File**: anie73128‐sup‐0001‐SuppMat.docx.

## Data Availability

The data that support the findings of this study are available from the corresponding author upon reasonable request.
