# Supplementary material for: Minute‐Scale Repeated Metabolic Probing of Living Cells Enabled by Rapid Parahydrogen‐Based Hyperpolarization
Source: Angew Chem Int Ed Engl. 2026 Jul 10;65(34):e4855536. doi: 10.1002/anie.4855536 (PMC13480487; doi:10.1002/anie.4855536)
Supplement: Supplementary file 1 [file ANIE-65-e4855536-s001.docx]

**Supporting Information**

**©Wiley-VCH 2021**

**69451 Weinheim, Germany**

**Supporting Information for:**

**Minute-scale repeated metabolic probing of living cells enabled by rapid parahydrogen-based hyperpolarization**

Philipp R. Groß^[a,b,c]^, Stefan Petersen^[a]^, Zirun Wang^[a]^, Behnam Shamshiri^[a]^, Henri de Maissin^[a,b]^, Sebastian Lucas^[d]^, Oliver Gorka^[e]^, Lisa Heß^[f]^, Irene Marco-Rius ^[g,h]^, Lluís Mangas-Florencio ^[g,h,i]^, André F. Martins^[j,k]^, Philipp Boehm-Sturm^[l,m,n]^, Maxim Zaitsev^[a]^, Robert Zeiser^[b,o]^, Jan-Bernd Hövener^[p]^, Thomas Reinheckel^[b,f,q]^, Olaf Groß^[b,e,r]^, Andreas B. Schmidt*^[a,b]^

Corresponding author: andreas.schmidt@uniklinik-freiburg.de

[a]	P. Groß, S. Petersen, H. de Maissin, Prof. Dr. M. Zaitsev, Dr. A. B. Schmidt
Division of Medical Physics, Department of Diagnostic and Interventional Radiology, University Medical Center Freiburg, Faculty of Medicine, University of Freiburg, Freiburg, Germany
E-mail: [andreas.schmidt@uniklinik-freiburg.de](mailto:andreas.schmidt@uniklinik-freiburg.de)

[b]	P. Groß, H. de Maissin, Prof. Dr. T. Reinheckel, Prof. Dr. O. Groß, Prof. Dr. R. Zeiser, Dr. A. B. Schmidt
German Cancer Consortium (DKTK), partner site Freiburg, a partnership between DKFZ and University Medical Center Freiburg, Im Neuenheimer Feld 280, Heidelberg 69120, Germany

[c]	P. Groß
Albert Ludwigs University Freiburg, Faculty of Biology, Schänzlestraße 1, Freiburg 79104, Germany

[d]	Dr. S. Lucas
NVision Imaging Technologies GmbH, Wolfgang-Paul-Str. 2, Ulm 89081, Germany

[e]	Prof. Dr. O. Groß, Dr. O. Gorka
Institute of Neuropathology, Faculty of Medicine, Medical Center, University of Freiburg, Freiburg, Germany

[f]	Dr. L. Heß, Prof. Dr. T. Reinheckel
Institute of Molecular Medicine and Cell Research, Faculty of Medicine, University of Freiburg, Stefan-Meier-Str. 17, Freiburg 79104, Germany

[g]	Dr. I. Marco-Rius, L. Mangas-Florencio

Institute for Bioengineering of Catalonia (IBEC), Barcelona Institute of Science and Technology (BIST), 08028 Barcelona, Spain

[h]	Dr. I. Marco-Rius, L. Mangas-Florencio

University of Barcelona, 08028 Barcelona, Spain

[i]	L. Mangas-Florencio

Vitala Technologies, S.L., 08028 Barcelona, Spain

[j]	Prof. Dr. A. F. Martins
Werner Siemens Imaging Center, Department of Preclinical Imaging and Radiopharmacy, Eberhard Karls University Tübingen, Röntgenweg 13, Tübingen 72076, Germany

[k]	Prof. Dr. A. F. Martins
Cluster of Excellence iFIT (EXC 2180) "Image-Guided and Functionally Instructed Tumor Therapies", Eberhard Karls University Tübingen

[l]	Prof. Dr. rer. nat. P. Boehm-Sturm

Charité – Universitätsmedizin Berlin, corporate member of Freie Universität Berlin and Humboldt-Universität zu Berlin, Charité 3R | Replace, Reduce, Refine, 10117 Berlin, Germany

[m]	Prof. Dr. rer. nat. P. Boehm-Sturm

Charité–Universitätsmedizin Berlin Department of Experimental Neurology and Center for Stroke Research Berlin, Charitéplatz 1, 10117 Berlin, Germany

[n]	Prof. Dr. rer. nat. P. Boehm-Sturm

Charité- Universitätsmedizin Berlin, Charité Core Facility Experimental MRIs (RRID SCR_017810), 10117 Berlin, Germany

[o]	Prof. Dr. R. Zeiser

Hematology and Oncology, Department of Medicine I, Medical Center, Faculty of Medicine, University of Freiburg, Hugstetter Strasse 55, Freiburg 79106, Germany

[p]	Prof. Dr. J.-B. Hövener

Sektion Biomedizinsche Bildgebung, Molecular Imaging North Competence Center MOINCC, Klinik für Radiologie und Neuroradiologie, University Hospital Schleswig-Holstein, Kiel University

[q]	Prof. Dr. T. Reinheckel
Centre for Biological Signalling Studies BIOSS, University of Freiburg, Freiburg 79104, Germany

[r]	Prof. Dr. O. Groß

Center for Integrative Biological Signalling Studies (CIBSS), University of Freiburg, Freiburg, Germany

**Table of Contents**

[**SABRE Hyperpolarization and dilution analysis**	2](#_Toc223535936)

[Sample Preparation	2](#_Toc223535937)

[Experimental Setup	3](#_Toc223535938)

[Polarization of [1-13C]Pyruvate and subsequent dilution	3](#_Toc223535939)

[**ICP Analysis of PBS diluted SABRE solution**	5](#_Toc223535940)

[**Cell Embedding and Bioreactor**	5](#_Toc223535941)

[Cell culture and Hydrogel Embedding of Cells	5](#_Toc223535942)

[Viability estimation of embedded cells	6](#_Toc223535943)

[DIY NMR compatible Bioreactor	7](#_Toc223535944)

[**13C NMR setup and acquisition**	9](#_Toc223535945)

[NMR Acquisitions and Data handling	9](#_Toc223535946)

[Active Region Measurement of 1T Benchtop NMR Spectrometer	10](#_Toc223535947)

[**Bibliography of SI**	11](#_Toc223535948)

# **SABRE Hyperpolarization and dilution analysis**

## Sample Preparation

Samples were prepared using 50 mM sodium [1-¹³C]pyruvate (Sigma-Aldrich), 6 mM IrIMes(COD)Cl precatalyst, 30 mM DMSO-d₆ (Sigma-Aldrich), and 0.65 mM EDTA in 600 µL methanol-d₄. Prior to mixing, the methanol-d₄ was degassed by bubbling N₂ through the solvent for approximately 2 min. The combined reagents were then sonicated for ~2 min and passed through a 1.2 µm syringe filter.

The IrIMes(COD)Cl precatalyst was synthesized based on a previously reported method^[1]^. In short: A vial was charged with IMes·HCl (60.9 mg, 0.178 mmol), [IrCl(cod)]₂ (60 mg, 0.089 mmol) and K₂CO₃ (74 mg, 0.534 mmol). The resulting mixture was suspended in acetone (3.0 mL) and stirred for 20 h at 60 °C. After this time the solvent was removed *in vacuo* and dichloromethane was added (3 mL). The mixture was filtered through a pad of silica. The pad of silica was washed with dichloromethane until the filtrate becomes colorless and the solvent was removed *in vacuo*. Pentane (3 mL) was added, the solution was sonicated for 1 min and kept in the fridge at −40 °C for 1 h. The precipitate was collected by filtration, washed with small amount of cold pentane and dried *in vacuo* to afford the desired complex as yellow-orange microcrystalline solid (76.8 mg, 67% yield).

Phosphate-buffered solutions (PBS) used during purification were prepared by dissolving commercially available phosphate buffer powder (pH 7.3–7.5, 0.017 g mL⁻¹, high purity; Sigma-Aldrich, SKU P7994-1EA) in deionized D₂O. Prior to usage, the PBS was degassed by N₂ bubbling for approximately 2 min.

Parahydrogen (≈90% enrichment) for the hyperpolarization experiments was generated using the setup described by Hövener et al.^[2]^

### Experimental Setup

The hyperpolarization was performed in a custom-built SABRE setup described in our previous paper ^[3]^establishing the SABRE-SHEATH conditions by generating a static sub-microtesla magnetic field along the z-axis (B_0_) using a resistive solenoid coil (360 mm length, 120 mm inner diameter, two layers of 1.12 mm copper wire, 47 compensation windings at each end of the solenoid). The B_0_ deviated by ± 1% over the sample volume (measured with a Fluxgate-Magnetometer Fluxmaster, Stefan-Mayer Instruments Germany). The hyperpolarization was performed in 5 mm NMR tubes (Wilmad Labglass) using a custom-built bubbling setup based on published designs ^[4–6]^ and a water bath regulating the sample temperature.

NMR measurements were carried out on a 1 T benchtop spectrometer (Spinsolve Carbon 43, Magritek) which is set to 38°C. Note, this spectrometer model allows for variable temperature control, a feature that is not available in newer spectrometers from the same vendor.

### Polarization of [1-13C]pyruvate and subsequent dilution

The SABRE solution, prepared as described in section ‘Sample preparation’, was polarized to up to 7.7 ± 0.2 % (Table S1) in 60 s using SABRE-SHEATH (B_0_ = 420 nT, T = 7 °C). After depressurizing the NMR tube, 50 µL of the hyperpolarized solution were withdrawn via the bubbling catheter using a 1 mL syringe. The extract was then diluted with 2.45 mL of degassed PBS/D_2_O (T ≈ 42 °C) in a magnetic field of approximately 120 mT, generated by a assembly of permanent magnets and steel plates sitting in a heated water bath to preheat the dilution buffer by rising steam. (Fig S1). The magnet assembly (permanent magnets and steel plates) was designed to avoid magnetic field zero crossings along the sample transfer path, ensuring a continuous elevated field during dilution and handling. Following ~8 s of active mixing by syringe aspiration and dispensing cycles, ~700 µL of the resulting solution were filtered, transferred into a NMR tube and measured in a 1T benchtop NMR. After dilution we recorded polarization levels of 5.26 ± 0.44 % (Table S1). The same procedure was used for the *in vitro* experiment where the NMR tube contained either embedded or suspended cells.

Figure S1: Magnet setup used to generate an elevated field during the dilution step. **a**: Preheating configuration of PBS in D_2_O prior to dilution, with the dilution vial open. **b**: Magnetic field strength within the vial, where the sample is located during mixing and dilution. **c**: Approximate positioning of the syringe used for dilution and subsequent filtration of the diluted SABRE solution. **d**: Magnetic field strength measured at the syringe position shown in c.

The ^13^C polarization was calculated by using a thermally polarized external ^13^C reference sample (neat [1-^13^C]acetic acid, CAS: 1563-79-7, 99 atom % ^13^C, Sigma Aldrich) using following equation:

$$P_{hyp}=\frac{I_{hyp}}{I_{ref}}\cdot\frac{c_{ref}}{c_{hyp}}\cdot\frac{N_{ref}}{N_{hyp}}\cdot\frac{f_{ref}}{f_{hyp}}\cdot P_{ref}$$

*I* denotes the integrated signal, *c* the concentration, *f* the ^13^C enrichment, and *N* the number of averages (for both measurements N = 1) of the hyperpolarized “hyp” or reference sample “ref”. *P*_ref_ is the thermal polarization of the reference sample at room temperature (298 K) and 1 T magnetic field of the NMR system.

Table S1: Compared concentrations of Methanol and [1-^13^C]pyruvate as well as Polarization of the [1-^13^C] in pyruvate before and after dilution of the SABRE solution 1:50 with PBS in D_2_O.

|  | Before dilution | After dilution (N=4) |
| --- | --- | --- |
| MeOH (M) | 24.7 | 0.74 ± 0.03 |
| Pyr (mM) | 50 | 1.3 ± 0.06 |
| P^13^C (%) | 7.7 ± 0.2 | 5.26 ± 0.44 |

# **ICP Analysis of PBS diluted SABRE solution**

The iridium concentration in all samples was quantified using an inductively coupled plasma–optical emission spectrometer (ICP-OES, PerkinElmer Avio 220 Max). Calibration was performed using a five-point external calibration curve. A single-element iridium standard solution (ROTI®Star ICP standard, 1000 mg L⁻¹ Ir in 10% HCl matrix; Carl Roth) was used to prepare the calibration standards. A matrix-matched blank was prepared following the same matrix composition as the samples to minimize potential matrix effects.

For sample preparation, a 500 µL aliquot of each sample was acidified with 1 mL of 67% HNO₃ (ICP-MS grade) and subsequently diluted to a final volume of 5 mL with deionized water (<5 MΩ·cm). Prior to analysis, all solutions were homogenized and allowed to equilibrate. The Ir emission line at 212.681 nm was selected for quantification based on its sensitivity and freedom from spectral interferences. Each sample was measured in triplicate to ensure analytical precision. The average Ir concentrations are displayed in Figure S1 below.

Figure S2: Iridium concentrations of SABRE solutions after 1:50 dilution with PBS and filtration through a 1.2 µm filter. Iridium concentrations were measured with ICP-MS (inductively coupled plasma mass spectroscopy) analysis.

# **Cell Embedding and Bioreactor**

### Cell culture and Hydrogel Embedding of Cells

Cultivation Conditions: HeLa were cultivated in T75 cell culture bottles using DMEM Medium (gibco 41966-029) supplemented with 10 % FCS (gibco A5256701) and 1 % Penicilin/Streptomycin (gibco 15140-122) at 37 °C and 5 % CO_2_ in an incubator until ~90 % confluency was achieved.

Splitting of cells: At ~90 % confluency, the medium was removed and the cells were washed once with 1X PBS (gibco 14190-094). 2 mL 0.05% Trypsin-EDTA (gibco 25300-054) was added and the cell culture bottle was incubated for 6 min. Cell culture medium was added and the cells were transferred into a new bottle 1:10.

Hydrogel embedding of cells: Prior to detaching the cells, 3 % (w/v) low melt agarose (Sigma Aldrich 2070-OP) in PBS was prepared by microwaving the needed amounts in a bottle. After complete dissolution of the agarose, the solution was autoclaved and subsequently aliquoted in 1 ml samples under a sterile bench. Furthermore, store bought sunflower oil was transferred into a glass bottle, autoclaved and stored cold until use. Upon preparation of agarose beads, an agarose aliquot was liquefied at 75 °C and stored in a custom-made aluminum rack in an incubator to equilibrate at 37 °C. HeLa from two fully confluent T75 were detached as described above and counted. After centrifugation, the cell pellet was resuspended with 10 µL culture Medium per 10^6^ cells, transferred into a 2 mL Eppendorf tube and put into the aluminum rack in the incubator to reach 37 °C. The rack was moved to a sterile bench and the suspension was quickly mixed with 5 µL low melt agarose per 10^6^ cells creating a mixture of about 1 % agarose. The mixture was drawn into a 1 mL syringe and dispensed into the oil through a 30G needle. The agarose was left to solidify for 1 min on ice. This resulted in agarose beads with an average diameter of 1.93 mm (N=23) and an average cell count of 2.5∙10^5^ cells per bead. The agarose beads were transferred into a 50 mL Falcon containing medium where the oil phase was discarded. The beads were transferred into a culture dish and washed multiple times with medium until the oil was removed. The beads were then stored in an incubator until further use.

### Viability estimation of embedded cells

To investigate the effect of the diluted SABRE solution on the embedded cells viability, the beads were produced as described above. After one night of incubation 20 beads per condition were transferred into a well of a 24 well plate. The medium was removed and 800 µL of prewarmed (37 °C) SABRE/PBS dilution of each condition were added to the according well. The multiwell plate was placed on a heat mat on a shaker for 3 min. Afterwards the diluted SABRE solution was replaced by cell medium for 5 min. This procedure was repeated two more times to simulate three injections in total. Additionally, beads that were kept in the bioreactor under perfusion at 37 °C overnight and beads that had received no treatment since production were added in separate wells after this step. Subsequently, the agarose beads were washed twice for 5 min each with pure 1X PBS on the heated shaker. For staining the washing PBS was replaced by 1X PBS containing 40 µg/mL Hoechst33342 and 40 µg/mL PI (Propidium iodide) and the samples were placed on the heated shaker, covered from light and incubated for 30 min. Following the staining, the beads were washed twice with pure 1X PBS as described above and subsequently imaged. The imaging was conducted on a Zeiss LSM710 confocal microscope and images were analyzed with a custom ImageJ script (Figure S2). The death rate was calculated as:

$$Death Rate in \%=100*\frac{Hoechst 33342 pos.}{Hoechst33342 pos. + Propidiumiodide pos.}$$

Figure S3: representative fluorescent-microscopy images of agarose embedded Hela after 3 treatments with methanol (b) or untreated (a). Blue (Hoechst33342 positive) cells were considered alive (positive), pink (Hoechst33342 positive and Propidiumiodide positive) where considered dead (negative) and red (Propidiumiodide positive) cells were also considered dead but as false positive.

An additional experiment was performed in which repeated injections of HP [1-^13^C]pyruvate to embedded HeLa where measured followed by a medium perfusion period of 15 min and an additional single injection of HP [1-^13^C]pyruvate (Figure S4). After an initial decline in the lactate-to-pyruvate ratio over the first three injections, the ratio returned to baseline levels following the perfusion period, indicating that no persistent or dominant impurity-related effect on metabolic conversion is present under these conditions.

Figure S4: **a**: Integrated ^13^C NMR signals of pyruvate (Pyr.) and lactate (Lac.) dynamically recorded after repeated injection of HP [1-^13^C]pyruvate into hydrogel-embedded HeLa cells (7,5 Mio) with ≈2 min intervals and after 15 minutes of medium circulation. The data for each injection was normalized to the respective maximum pyruvate signal to support comparing the lactate dynamics. **b**: the lactate-to-pyruvate AUC ratios derived from the dynamic data shown in panel a.

### DIY NMR compatible Bioreactor

The design of the Bioreactor (BR) was inspired by previously published work^[7,8]^ and adapted to fit our hardware and workflow. It consists of two custom designed and 3-D printed parts, as well as commercially available parts. All parts are listed in **Fehler! Verweisquelle konnte nicht gefunden werden.** and the assembly is displayed in **Fehler! Verweisquelle konnte nicht gefunden werden.**2. The BR in its working setup (connected to two peristaltic pumps) is displayed in Figure S3. The inlet tubes and connector were chosen with small inner diameters (ID) to reduce dead volume. The outlet tubes were chosen with bigger IDs to ensure sufficient removal of fluid and prevent clogging. The BR was designed in a way to keep the embedded cells in the active volume of the NMR when used with 5 mm NMR tubes.

Table S2: Parts used to construct the Bioreactor

| Part | Manufacturer | Part Number |
| --- | --- | --- |
| Bioreactor Top | 3D printed in house | - |
| Bioreactor Plug | 3D printed in house | - |
| Peristaltic Pumps | Drifton | LP-BQ50-1JWA |
| Inlet Peristaltic Tubing | Drifton | LP-PLATINUM-0.5*0.8 |
| Outlet Peristaltic Tubing | Drifton | LP-PLATINUM-3*1 |
| Inlet Reactor Tubings | Postnova  SCP | FS-115  45.159.025.00 |
| Outlet Reactor Tubings | SCP | 45.159.100.00 |
| Connecting Tubing | SCP | 42.318.159.00 |
| Tubing Connectors | Postnova  SMC | P-702, P703, P-882, P-652  KQ2H |
| Luer Lock Parts | Postnova | P-604, P-655 |
| 5 mm NMR tube | Norell | ST500-8 |

Figure S5: Bioreactor and it’s components. **a**.: Lower assembly of the BR displaying the yellow 360 µm ID inlet catheter for medium perfusion and for injection of hyperpolarized solutions as well as the lower end of the outlet catheter for solution removal. In white the 3D printed BR Plug can be seen which is secured to the outlet tube using shrink tube. **b**.: lower assembly of the BR inside a 5 mm NMR tube. **c**.: Upper assembly of the BR shown in the medium perfusion mode with the 3D printed BR Top in black as well as tubing connectors. **d**.: Upper assembly of the BR shown in the injection mode with a 1 mL luer lock syringe attached. **e**.: BR completely assembled in the depth gauge for use with the 1T Magritek Benchtop spectrometer. **f**.: Lower assembly of the BR with 5 mm NMR tube in the depth gauge showing the placement of the BR volume in the active volume of the spectrometer (identified in Figure S6 and marked by tapes). g: lower assembly of the BR in experiment setting, with HeLa containing agarose beads inside. f: lower assembly of the BR in experiment setting within the depth gauge showing that the agarose beads are held within the active volume (between the tapes).

Figure S6: The bioreactor setup in operation. Depicted is the bioreactor placed in a 1T benchtop NMR system and connected to two peristaltic pumps (medium inlet and waste outlet).

# **13C NMR setup and acquisition**

### NMR Acquisitions and Data handling

All NMR acquisitions were performed on a 1 T benchtop NMR spectrometer (Spinsolve Carbon 43, Magritek) that was set to 38 °C bore temperature. For dynamic acquisitions, a series of 20° pulses was used with a repetition time of ~6 s. The data was handled using a NMR Glue based Python script and visualized using Origin Pro 2024 (Version 10.1.0.178).

^13^C NMR data corresponding to data shown in main text Figure 3 is displayed in Figure S4.

Figure S7: Waterfall plots of dynamic NMR acquisitions after injection of HP-Pyruvate to 7.5 Mio agarose embedded HeLa cells. a. multiple injections to the same sample within ~2 min intervals and no intermediate cell culture medium exposure. b. multiple injections to the same sample within ~20 min intervals and 15 min intermediate cell culture medium exposure.

### Active Region Measurement of 1T Benchtop NMR Spectrometer

To properly record NMR signal from cells in the NMR tube, be it in suspension mixed with hyperpolarized solution or hydrogel embedded, they must be in the active region of the NMR spectrometer. To characterize the active region of the spectrometer we used a 5 mm NMR tube that was increasingly filled with water and each time measured using a 90° pulse. The resulting spectra were then integrated and the area under the curve (AUC) plotted against the fill height of the tube (Figure S5). Using this data, we concluded the active are to be between 20 mm and 40 mm fill height.

Figure S8: Normalized integrated water signal from a variably filled 5mm NMR tube. Recorded on a Spinsolve Carbon 43, Magritek

# **Bibliography of SI**

[1]	R. Savka, H. Plenio, “Facile synthesis of [(NHC)MX(cod)] and [(NHC)MCl(CO)2] (M = Rh, Ir; X = Cl, I) complexes” *Dalton Trans.* **2014**, *44*, 891–893.

[2]	J.-B. Hövener, S. Bär, J. Leupold, K. Jenne, D. Leibfritz, J. Hennig, S. B. Duckett, D. von Elverfeldt, “A continuous-flow, high-throughput, high-pressure parahydrogen converter for hyperpolarization in a clinical setting” *NMR Biomed* **2013**, *26*, 124–131.

[3]	H. de Maissin, P. R. Groß, O. Mohiuddin, M. Weigt, L. Nagel, M. Herzog, Z. Wang, R. Willing, W. Reichardt, M. Pichotka, L. Heß, T. Reinheckel, H. J. Jessen, R. Zeiser, M. Bock, D. von Elverfeldt, M. Zaitsev, S. Korchak, S. Glöggler, J.-B. Hövener, E. Y. Chekmenev, F. Schilling, S. Knecht, A. B. Schmidt, “In Vivo Metabolic Imaging of [1-13C]Pyruvate-d3 Hyperpolarized By Reversible Exchange With Parahydrogen” *Angewandte Chemie International Edition* **2023**, *n/a*, e202306654.

[4]	S. Nantogma, M. R. H. Chowdhury, M. S. H. Kabir, I. Adelabu, S. M. Joshi, A. Samoilenko, H. de Maissin, A. B. Schmidt, P. Nikolaou, Y. A. Chekmenev, O. G. Salnikov, N. V. Chukanov, I. V. Koptyug, B. M. Goodson, E. Y. Chekmenev, “MATRESHCA: Microtesla Apparatus for Transfer of Resonance Enhancement of Spin Hyperpolarization via Chemical Exchange and Addition” *Anal Chem* **2024**, *96*, 4171–4179.

[5]	A. B. Schmidt, H. de Maissin, I. Adelabu, S. Nantogma, J. Ettedgui, P. TomHon, B. M. Goodson, T. Theis, E. Y. Chekmenev, “Catalyst-Free Aqueous Hyperpolarized [1-13C]Pyruvate Obtained by Re-Dissolution Signal Amplification by Reversible Exchange” *ACS Sens.* **2022**, *7*, 3430–3439.

[6]	A. S. Kiryutin, G. Sauer, S. Hadjiali, A. V. Yurkovskaya, H. Breitzke, G. Buntkowsky, “A highly versatile automatized setup for quantitative measurements of PHIP enhancements” *J Magn Reson* **2017**, *285*, 26–36.

[7]	J. Carvalho, S. Alves, M. M. C. A. Castro, C. F. G. C. Geraldes, J. A. Queiroz, C. P. Fonseca, C. Cruz, “Development of a bioreactor system for cytotoxic evaluation of pharmacological compounds in living cells using NMR spectroscopy” *Journal of Pharmacological and Toxicological Methods* **2019**, *95*, 70–78.

[8]	L. Mangas-Florencio, A. Herrero-Gómez, J. Eills, M. Azagra, M. Batlló-Rius, I. Marco-Rius, “A DIY Bioreactor for in Situ Metabolic Tracking in 3D Cell Models via Hyperpolarized 13C NMR Spectroscopy” *Anal. Chem.* **2025**, DOI 10.1021/acs.analchem.4c04183.
